# Are hypertensive disorders in pregnancy associated with congenital malformations in offspring? Evidence from the WHO Multicountry cross sectional survey on maternal and newborn health

**DOI:** 10.1186/s12884-016-0987-8

**Published:** 2016-07-29

**Authors:** S. Bellizzi, M. M. Ali, E. Abalos, A. P. Betran, J. Kapila, C. Pileggi-Castro, J. P. Vogel, M. Merialdi

**Affiliations:** 1 World Health Organization, Eastern Mediterranean Regional Office, P.O. Box 7608, Nasr City, Cairo, 11371 Egypt; 2 Centro Rosarino de Estudios Perinatales (CREP) Moreno 878, 6° Piso. (S2000DKR), Rosario, Argentina; 3 Department of Reproductive Health and Research, World Health Organization, UNDP/UNFPA/UNICEF/WHO/World Bank Special Programme of Research, Development and Research Training in Human Reproduction (HRP), Avenue Appia 20, Geneva, Switzerland; 4 Maternal & Child Morbidity & Mortality Surveillance Unit, Family Health Bureau - Ministry of Health, 231 De Saram Place, Colombo, 10 Sri Lanka; 5 Department of Pediatrics, Ribeirão Preto Medical School, University of São Paulo, Ribeirão Preto, Brazil; 6 BD. 1 Becton Drive, MC 374, Franklin Lakes, NJ 07417-1885 USA

## Abstract

**Background:**

Annually, around 7.9 million children are born with birth defects and the contribution of congenital malformations to neonatal mortality is generally high. Congenital malformations in children born to mothers with hypertensive disorders during pregnancy has marginally been explored.

**Methods:**

Country incidence of congenital malformations was estimated using data on the 310 401 livebirths of the WHO Multicountry Survey which reported information from 359 facilities across 29 countries. A random-effect logistic regression model was utilized to explore the associations between six broad categories of congenital malformations and the four maternal hypertensive disorders “Chronic Hypertension”, “Preeclampsia” and “Eclampsia” and “Chronic hypertension with superimposed preeclampsia”.

**Results:**

The occupied territories of Palestine presented the highest rates in all groups of malformation except for the “Lip/Cleft/Palate” category. Newborns of women with chronic maternal hypertension were associated with a 3.7 (95 % CI 1.3–10.7), 3.9 (95 % CI 1.7–9.0) and 4.2 (95 % CI 1.5–11.6) times increase in odds of renal, limb and lip/cleft/palate malformations respectively. Chronic hypertension with superimposed preeclampsia was associated with a 4.3 (95 % CI 1.3–14.4), 8.7 (95 % CI 2.5–30.2), 7.1 (95 % CI 2.1–23.5) and 8.2 (95 % CI 2.0–34.3) times increase in odds of neural tube/central nervous system, renal, limb and Lip/Cleft/Palate malformations.

**Conclusions:**

This study shows that chronic hypertension in the maternal period exposes newborns to a significant risk of developing renal, limb and lip/cleft/palate congenital malformations, and the risk is further exacerbate by superimposing eclampsia. Additional research is needed to identify shared pathways of maternal hypertensive disorders and congenital malformations.

## Background

Annually, around 7.9 million children are born with birth defects [1]. At least 3.3 million children under 5 years of age die from birth defects every year and an estimated 3.2 million of those who survive may be disabled for life [1]. The contribution of congenital malformations to neonatal mortality is generally higher, in lower infant mortality countries [1].

Eclampsia/pre-eclampsia syndrome consists of a state of excessive systemic inflammation causing new-onset proteinuria and hypertension during the second half of pregnancy [2]. Pre-eclampsia affects between two and eight percent [3–7] of all pregnancies with a worldwide estimation of 8 370 000 cases per year whilst eclampsia ranges from 0.3 to 1.4 % [6, 7]. The syndrome affects both the mother and her fetus and the pathogenesis features an impaired placental perfusion and widespread endothelial cell dysfunction [8, 9]. Severe pre-eclampsia is a major cause of severe maternal/fetal morbidity and adverse perinatal outcomes, such as prematurity and intrauterine growth restriction [10].

Only a few studies have explored the associations between pre-eclampsia and malformations providing inconclusive results: one reported an increased risk of renal dysgenesis (OR 4.7, 95 % CI 1.7–12.8), esophageal atresia/stenosis (OR 4.6, 95 % CI 1.8–12.2) and rectal/anal stenosis (OR 3.7, 95 % CI 1.6–8.5) in the offspring of pregnant women who developed preeclampsia with superimposed chronic hypertension [11] whilst another found that esophageal atresia/stenosis was a greater risk in pregnant women with chronic hypertension (OR 3.1, 95 % CI 1.4–6.8) [12]. Some studies have suggested a correlation between maternal hypertension and severe hypospadias (OR 2.1, 95 % CI 1.6–2.9) [13, 14]. Altered perfusion of placenta and embryo/fetus is being considered as plausible biological pathway [15]; however, there is a dearth of knowledge on the likely common events leading to hypertensive disorders and congenital abnormalities [16] mainly because of the different gestational timing of these two separate events, namely first trimester of gestation for congenital malformation and second/third trimester for hypertensive disorders [17]. Using data collected in 29 countries worldwide as part of the World Health Organization Multicountry Survey (WHOMCS), in this analysis we aimed to examine the association between hypertensive disorders of pregnancy and the risk of congenital malformations in the newborn.

## Methods

### Settings and participants

The study population and data collection methods used in this survey are described in detailed elsewhere [18]. In brief, the WHOMCS was an international, multi-country, cross-sectional survey for all delivering mothers and their newborns in 359 facilities across 29 countries involving over 1500 collaborators. It was conducted from May 2010 to December 2011 and captured data from over 314 000 deliveries.

The Study involved five WHO regions: African Region (Angola, DR Congo, Kenya, Niger, Nigeria and Uganda); Region of the Americas (Argentina, Brazil, Ecuador, Mexico, Nicaragua, Paraguay and Peru); Eastern Mediterranean Region (Afghanistan, Jordan, Lebanon, occupied Palestinian territory, Palestine, Pakistan and Qatar); South-East Asia Region (India, Nepal, Sri Lanka and Thailand); Western Pacific Region (Cambodia, China, Japan, Mongolia, Philippines and Vietnam).

The hospitals with a minimum of 1000 deliveries per year were identified. Within each country, the capital city was included, along with two randomly selected provinces with probability proportional to their population size. In each province and in the capital city, seven hospitals with over 1000 deliveries per year and the capacity to perform caesarean sections were selected using a multi-stage cluster sampling method.

Participants in the study were all women giving birth during the data collection period in the participating hospitals together with their respective newborns, all maternal near-miss cases and all maternal deaths taking place in the participating hospitals up to seven postpartum/postabortion days, discharge or death (whichever came first), regardless of the gestational age and delivery status.

Data were collected from hospital medical records through daily visits to the obstetrical/postpartum ward, gynecologic/abortion care unit, delivery room and intensive care unit for the duration of 2 months if the hospital had 6000 deliveries/year or and three months if the health facility had less than 6000 deliveries/year [19].

### Study population

In the WHOMCS, ascertainment of malformations was made by obstetricians and pediatrician and the inclusion criteria were major congenital anomalies among live births and stillbirths.

All live births (*n* = 310 401) during the data collection were included to explore the frequency of congenital malformation.

We aimed to evaluate the risk of specific categories of congenital malformation against maternal hypertensive disorders. Multiple pregnancies were excluded from the analysis since more susceptible to maternal complications, potentially distorting estimates. Also stillbirths were excluded from the analysis, as our preliminary analysis indicated that congenital malformations were significantly under-reported in stillbirths.

**Fig. 1:**
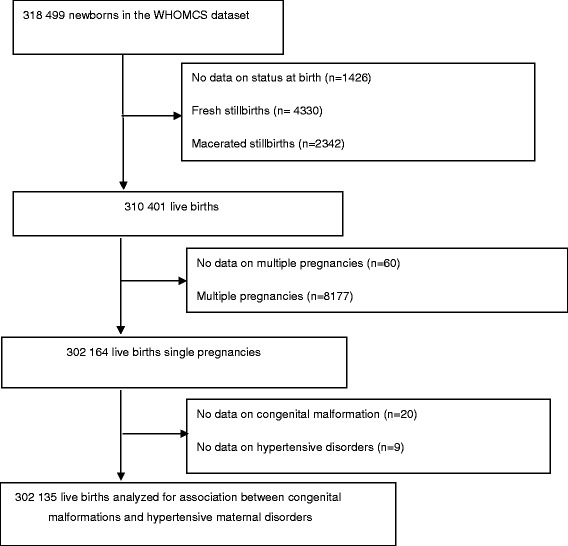
Study population selection process

Mothers and newborns with missing information on status at birth (*n* = 1426) or number of fetuses (*n* = 60) were excluded. Individuals with missing values on congenital malformations (*n* = 20) and maternal hypertensive disorders (*n* = 9) were also excluded in the analysis leaving a final study population of 302 135 women. See flowchart showing the selection of study participants in Fig. 1.

### Outcomes and exposures

In the WHOMCS, data collectors performed daily visits to the obstetrical/postpartum ward, gynaecologic/abortion care unit, delivery room and intensive care unit to review medical records and extract data from these into individual data forms.

Six categories of congenital malformations were defined for data collector to extract data from the medical records in line with the International Clearinghouse for Birth Defects Monitoring Systems classification [20].

The six groups consisted of: “Neural tube/Central Nervous System” (Anencephaly, Spina Bifida, Encephalocele, Microcephaly, Holoprosencephaly, Hydrocephaly, Anophthalmos/microphtalmos, Anotia/microtia) “Cardiac” (Transposition of great vessels, Tetralogy of Fallot, Hypoplastic left Heart Syndrome, Coarctation of the aorta), “Renal” (Renal agenesis, Cystic kidney, Bladder exstrophy), “Limb” (Polydactyly, Limb reduction defects), “Lip/Cleft/Palate” (Choanal atresia, Cleft palate without cleft lip, Cleft lip with or without cleft palate) and “Chromosomal” (Trisomy 13, Trisomy 18, Down syndrome).

Hypertensive disorders information was collected from delivery records and was categorized into “Chronic Hypertension”, “Preeclampsia” and “Eclampsia”. The study definition for pre-eclampsia was the presence of hypertension (blood pressure >140/90 mmHg) associated with proteinuria in women known to be previously normotensive. Eclampsia was defined as the occurrence of convulsions and/or coma unrelated to other cerebral conditions in women with signs and symptoms of pre-eclampsia. Seizures are of grand mal type and may first appear before labour, during labour or up to 48 h postpartum. Chronic hypertension was defined as a blood pressure >140/90 mmHg diagnosed prior to the onset of pregnancy or before the 20th week of gestation; all women with chronic hypertension and who developed preeclampsia were grouped and classified under the new variable “Chronic hypertension with superimposed preeclampsia”.

### Statistical analysis

We determined the frequency of newborns with congenital malformations per 1000 live births for each of the 29 countries participating in the WHOMCS stratified by WHO Region and by Human Development Index (HDI) [21]. Whereas the categorization by WHO regions gives a geographical perspective, it can conceal important epidemiological differences and patterns. HDI is a summary measure of human development. It measures the average achievements in a country in three dimensions of human development: a long and healthy life, access to knowledge and a decent standard of living. Categorization of countries by HDI is an increasingly used approach as it pools together more similar countries [22, 23].

We used frequencies to describe the congenital malformation categories by maternal age, marital status, woman education and number of previous births; as health facilities were the primary sampling unit of the WHOMCS, individual-level analyses may be affected by clustering. After excluding missing data, all estimates of association (chi-square tests) were corrected for the cluster effects (health facilities as sampling units, countries as strata) and *P* < 0.05 was regarded as significant.

Crude and adjusted ORs with 95 % CI were evaluated using a multi-category logistic regression models for the occurrence of any congenital malformation; the adjusted logistic regression model considered potential maternal confounders: age, marital status, woman education and number of previous births. Because of the possible cluster effect of individual analysis [24] we generated the OR adjusted for the random mixed effect of facilities nested within countries and took into account the sampling selection of the additional provinces into account. Considering the large variability of reporting amongst WHO Regions and specifically the very low incidence of congenital malformation reported in the African Region participating countries, we performed a sensitivity analysis excluding the African Region countries.

Stata 13 was used for statistical analyses and p-values <0.05 was regarded as significant.

The WHOMCS was approved by the WHO Ethical Review Committee and the relevant ethical clearance bodies in participating countries and facilities. Written consent from individual participants was not required.

## Results

In the WHOMCS, overall 1706 newborns with congenital malformations were recorded, distributed as follows: 322 (1.04 per 1000 livebirths) “Neural tube/Central Nervous System”, 494 (1.59 per 1000 livebirths) “Cardiac”, 136 (0.44 per 1000 livebirths) “Renal”, 367 (1.18 per 1000 livebirths) “Limb”, 212 (0.68 per 1000 livebirths) “Lip/Cleft/Palate” and 175 (0.56 per 1000 livebirths) “Chromosomal”.

By country, the occupied territories of Palestine presented the highest rates in all malformation except for “Lip/Cleft/Palate” where Japan was highest. In the occupied territories of Palestine, it was found a prevalence of 10.2 cases of “Neural tube/Central Nervous System”, 24.6 cases of “Cardiac”, 11.3 cases for “Renal”, 8.2 cases for “Limb” and 3.1 cases for “Chromosomal” (Table 1).

**Table 1:** The prevalence of newborn with congenital malformations by country per 1000 livebirths

WHO Region	Country	Hospitals	Live births	Neur.tube/CNS defects		Cardiac defects		Renal defects		Limb defects		Lip/cleft/palate defects		Chromosomal syndrome		Total congenital defects	
AFR	Angola	20	10 072	0	0	1	0.1	0	0	1	0.1	1	0.1	1	0.1	4	0.4
	DR Congo	21	8 525	5	0.6	1	0.1	0	0	6	0.7	2	0.2	1	0.1	15	1.7
	Kenya	20	20 057	30	1.5	6	0.3	2	0.1	55	2.7	18	0.9	11	0.5	122	6.1
	Niger	11	10 714	0	0	0	0	0	0	2	0.2	0	0	0	0	2	0.2
	Nigeria	21	12 094	8	0.7	2	0.2	2	0.2	4	0.3	4	0.3	1	0.1	21	1.7
	Uganda	20	10 626	1	0.1	0	0	0	0	10	0.9	2	0.2	1	0.1	14	1.3
	Total	113	72 088	44	0.6	10	0.1	4	0.1	78	1.1	27	0.4	15	0.2	178	2.5
AMR	Argentina	14	9 851	14	1.4	25	2.5	8	0.8	10	1.0	5	0.5	14	1.4	76	7.7
	Brazil	7	7 093	6	0.8	5	0.7	3	0.4	6	0.8	7	1.0	6	0.8	33	4.6
	Ecuador	18	10 124	2	0.2	14	1.4	2	0.2	4	0.4	3	0.3	8	0.8	31	3.1
	Mexico	14	13 312	31	2.3	39	2.9	18	1.3	33	2.5	12	0.9	13	1.0	146	11.0
	Nicaragua	8	6 488	11	1.7	6	0.9	3	0.5	12	1.8	8	1.2	7	1.1	47	7.2
	Paraguay	6	3 626	2	0.6	4	1.1	2	0.6	1	0.3	0	0	1	0.3	10	2.7
	Peru	16	15 203	28	1.8	126	8.3	14	0.9	31	2.0	22	1.4	40	2.6	277	18.2
	Total	83	65 697	94	1.4	219	3.3	50	0.8	97	1.5	57	0.9	89	1.3	606	9.2
EMR	Afghanistan	8	24 478	25	1.0	0	0	2	0.1	5	0.2	4	0.2	2	0.1	38	1.5
	Jordan	1	1 197	3	2.5	7	5.8	3	2.5	2	1.7	6	5.0	3	2.5	24	20.0
	Lebanon	9	4 118	9	2.2	21	5.1	3	0.7	0	0	1	0.2	3	0.7	37	9.0
	OPT	1	975	10	10.2	24	24.6	11	11.3	8	8.2	1	1.0	3	3.1	57	58.5
	Pakistan	16	12 889	38	2.9	7	0.5	13	1.0	20	1.6	13	1.0	4	0.3	95	7.4
	Qatar	1	4 000	4	1.0	13	3.3	9	2.2	2	0.5	2	0.5	4	1.0	34	8.5
	Total	36	48 657	89	1.8	72	1.5	41	0.8	37	0.8	27	0.5	19	0.4	285	5.8
SEAR	India	21	30 399	34	1.1	14	0.5	19	0.6	29	0.9	11	0.4	7	0.2	114	3.7
	Nepal	8	11 117	8	0∙7	3	0.3	0	0	2	0.2	4	0.4	1	0.1	18	1.6
	Sri Lanka	14	18 162	22	1.2	88	4.8	10	0.6	75	4.1	32	1.8	24	1.3	251	13.8
	Thailand	12	8 976	13	1.5	17	1.9	5	0.6	5	0.6	17	1.9	7	0.8	64	7.1
	Total	55	68 654	77	1.1	122	1.8	34	0.5	111	1.6	64	0.9	39	0.6	447	6.5
WPR	Cambodia	5	4 691	0	0	2	0.4	0	0	2	0.4	2	0.4	1	0.2	7	1.5
	China	21	13 395	3	0.2	22	1.6	2	0.1	23	1.7	14	1.0	0	0	64	4.8
	Japan	10	3 566	2	0.6	35	9.8	2	0.6	5	1.4	8	2.2	7	2.0	59	16.5
	Mongolia	5	7 385	1	0.1	6	0.8	0	0	5	0.7	2	0.3	2	0.3	16	2.2
	Philippines	16	10 711	9	0.8	1	0.1	1	0.1	6	0.6	9	0.8	1	0.1	27	2.5
	Viet Nam	15	15 557	3	0.2	5	0.3	2	0.1	3	0.2	2	0.1	2	0.1	17	1.1
	Total	72	55 305	18	0.3	71	1.3	7	0.1	44	0.8	37	0.7	13	0.2	190	3.4
Overall Countries		359	310 401	322	1.0	494	1.6	136	0.4	367	1.2	212	0.7	175	0.6	1 706	5.5

On the other hand, Niger reported only two cases of “Limb”, with a total of 0.2 malformation per 1000 live births.

When considering prevalence of congenital malformations by WHO Regions the highest prevalence of malformations as a whole was reported in the Region of the Americas with 9.2 cases per 1000 live births and the lowest in the African Region with 2.5 cases for 1000 live births (Table 1).

The stratification by HDI showed a clear linear trend as countries with high HDI and very high HDI had the highest frequency of congenital malformations (11.4 per 1000 livebirths and 9.7 per 1000 livebirths respectively) followed countries with medium HDI (4.3 per 1000 livebirths), and finally countries with low HDI (2.7 per 1000 livebirths) (Table 2).

**Table 2:** The prevalence of newborn with congenital malformations by HDI quartiles per 1000 livebirths

HDI	Country	Hospitals	Live births	Neur.tube/CNS defects		Cardiac defects		Renal defects		Limb defects		Lip/cleft/palate defects		Chromosomal syndrome		Total congenital defects	
Very High	Argentina	14	9 851	14	1.4	25	2.5	8	0.8	10	1.0	5	0.5	14	1.4	76	7.7
	Japan	10	3 566	2	0.6	35	9.8	2	0.6	5	1.4	8	2.2	7	2.0	59	16.5
	Qatar	1	4 000	4	1.0	13	3.3	9	2.2	2	0.5	2	0.5	4	1.0	34	8.5
	Total	25	17 417	20	1.1	73	4.2	10	1.1	17	1.0	15	0.9	25	1.4	169	9.7
High	Brazil	7	7 093	6	0.8	5	0.7	3	0.4	6	0.8	7	1.0	6	0.8	33	4.6
	Ecuador	18	10 124	2	0.2	14	1.4	2	0.2	4	0.4	3	0.3	8	0.8	31	3.1
	Lebanon	9	4 118	9	2.2	21	5.1	3	0.7	0	0	1	0.2	3	0.7	37	9.0
	Mexico	14	13 312	31	2.3	39	2.9	18	1.3	33	2.5	12	0.9	13	1.0	146	11.0
	Peru	16	15 203	28	1.8	126	8.3	14	0.9	31	2.0	22	1.4	40	2.6	277	18.2
	Sri Lanka	14	18 162	22	1.2	88	4.8	10	0.6	75	4.1	32	1.8	24	1.3	251	13.8
	Total	78	68 012	98	1.1	293	4.3	50	0.7	149	2.2	77	1.1	94	1.4	775	11.4
Medium	India	21	30 399	34	1.1	14	0.5	19	0.6	29	0.9	11	0.4	7	0.2	114	3.7
	Jordan	1	1 197	3	2.5	7	5.8	3	2.5	2	1.7	6	5.0	3	2.5	24	20.0
	Cambodia	5	4 691	0	0	2	0.4	0	0	2	0.4	2	0.4	1	0.2	7	1.5
	China	21	13 395	3	0.2	22	1.6	2	0.1	23	1.7	14	1.0	0	0	64	4.8
	Mongolia	5	7 385	1	0.1	6	0.8	0	0	5	0.7	2	0.3	2	0.3	16	2.2
	Nicaragua	8	6 488	11	1.7	6	0.9	3	0.5	12	1.8	8	1.2	7	1.1	47	7.2
	OPT	1	975	10	10.2	24	24.6	11	11.3	8	8.2	1	1.0	3	3.1	57	58.5
	Paraguay	6	3 626	2	0.6	4	1.1	2	0.6	1	0.3	0	0	1	0.3	10	2.7
	Philippines	16	10 711	9	0.8	1	0.1	1	0.1	6	0.6	9	0.8	1	0.1	27	2.5
	Thailand	12	8 976	13	1.5	17	1.9	5	0.6	5	0.6	17	1.9	7	0.8	64	7.1
	Viet Nam	15	15 557	3	0.2	5	0.3	2	0.1	3	0.2	2	0.1	2	0.1	17	1.1
	Total	111	103 400	89	1.0	108	1.0	48	0.5	96	0.9	72	0.7	34	0.3	447	4.3
Low	Afghanistan	8	24 478	25	1.0	0	0	2	0.1	5	0.2	4	0.2	2	0.1	38	1.5
	Angola	20	10 072	0	0	1	0.1	0	0	1	0.1	1	0.1	1	0.1	4	0.4
	DR Congo	21	8 525	5	0.6	1	0.1	0	0	6	0.7	2	0.2	1	0.1	15	1.7
	Kenya	20	20 057	30	1.5	6	0.3	2	0.1	55	2.7	18	0.9	11	0.5	122	6.1
	Nepal	8	11 117	8	0.7	3	0.3	0	0	2	0.2	4	0.4	1	0.1	18	1.6
	Niger	11	10 714	0	0	0	0	0	0	2	0.2	0	0	0	0	2	0.2
	Nigeria	21	12 094	8	0.7	2	0.2	2	0.2	4	0.3	4	0.3	1	0.1	21	1.7
	Pakistan	16	12 889	38	2.9	7	0.5	13	1.0	20	1.6	13	1.0	4	0.3	95	7.4
	Uganda	20	10 626	1	0.1	0	0	0	0	10	0.9	2	0.2	1	0.1	14	1.3
	Total	145	121 572	115	1.2	20	0.2	19	0.2	105	0.9	48	0.4	22	0.2	329	2.7
Overall Countries		359	310 401	322	1.0	494	1.6	136	0.4	367	1.2	212	0.7	175	0.6	1 706	5.5

We noted that “Chromosomal” malformations were significantly higher with increasing maternal age and increasing number of previous births; similarly “Cardiac” malformations were significantly more common as maternal age and years of education increase (Table 3).

**Table 3:** Maternal characteristics in all Congenital Malformations groups

	All women	Neural tube/Central nervous system		Cardiac		Renal		Limb		Lip/Cleft/Palate		Chromosomal	
	N	n (%)	*χ* ^2^	n (%)	*χ* ^2^	n (%)	*χ* ^2^	n (%)	*χ* ^2^	n (%)	*χ* ^2^	n (%)	*χ* ^2^
Maternal age (years)			0.9		<0.01		0.07		0.5		0.9		<0.01
<20	31 259	31 (9.8)		39 (8.2)		7 (5.2)		42 (11.8)		18 (8.5)		14 (8.1)	
20–35	244 425	238 (75.5)		340 (71.4)		111 (82.8)		278 (78.5)		162 (76.8)		91 (52.9)	
>35	25 583	46 (14.7)		97 (20.4)		16 (12.0)		34 (9.7)		31 (14.7)		67 (39.0)	
Missing	868												
Marital Status			0.6		0.09		0.3		0.7		0.09		0.5
Single	30 583	35 (11.2)		32 (6.8)		10 (7.5)		38 (10.8)		12 (5.8)		15 (8.8)	
Married	268 051	278 (88.8)		440 (93.2)		124 (92.5)		314 (89.2)		194 (94.2)		155 (91.2)	
Missing	3 501												
Education (years)			0.4		<0.01		0.9		<0.01		<0.01		0.8
<5	54 693	55 (18.5)		13 (3.2)		13 (10.1)		38 (12.4)		16 (8.6)		13 (8.8)	
5–8	62 952	68 (23.0)		60 (14.8)		22 (17.0)		70 (22.9)		41 (21.8)		24 (16.3)	
9–11	71 550	88 (29.7)		166 (41.0)		40 (31.0)		105 (34.3)		77 (40.9)		53 (36.1)	
>11	88 274	85 (28.8)		165 (41.0)		54 (41.9)		93 (30.4)		54 (28.7)		57 (38.8)	
Missing	24 666												
N previous births			0.3		0.2		0.5		0.4		0.2		<0.01
0	128 479	123 (39.0)		208 (43.6)		52 (38.5)		161 (45.2)		76 (36.2)		58 (33.5)	
1–2	125 401	135 (42.9)		216 (45.3)		63 (46.7)		156 (43.8)		97 (46.2)		78 (45.1)	
>2	47 672	57 (18.1)		53 (11.1)		20 (14.8)		39 (11.0)		37 (17.6)		37 (21.4)	
Missing	583												

The study population included 1152 women with chronic hypertension, 6163 women with preeclampsia, 765 women with eclampsia and 294 with preeclampsia superimposed on chronic hypertension.

Some variability on reporting exposure data existed across WHO regions: chronic hypertension incidence ranged from 2.3 cases per 1000 livebirths in SEAR to 6.6 cases per 1000 livebirths in AMR; preeclampsia was the lowest in EMR with 12.1 cases per 1000 livebirths and highest in AMR with 38.2 cases per 1000 livebirths; eclampsia varied from a low 1.2 cases per 1000 livebirths in WPR to 5.0 cases each 1000 livebirths in AFR; finally chronic hypertension with superimposed preeclampsia ranged from 0.2 cases per 1000 livebirths in SEAR to 2.1 per 1000 livebirths in AMR.

Table 4 presents the crude and adjusted odd ratios for the four the different categories of hypertensive disorders of pregnancy and the different birth defects considered in this analysis.

**Table 4:** Crude and adjusted Odds Ratio of pregnancy hypertensive disorders and congenital malformations

	Chronic Hypertension		Pre-eclampsia		Eclampsia		Chronic HT/preeclampsia	
	Crude OR (CI 95 %)	Adj OR^a^ (CI 95 %)	Crude OR (CI 95 %)	Adj OR^a^ (CI 95 %)	Crude OR (CI 95 %)	Adj OR^a^ (CI 95 %)	Crude OR (CI 95 %)	Adj OR^a^ (CI 95 %)
Congenital malformation								
Neural tube/Central Nervous System	4.2 (1.7–10.2)	2.3 (0.9–5∙.9)	2.0 (1.2–3.6)	1.5 (0.8–2.7)	-	-	9.9 (3.1–31.0)	4.3 (1.3–14.4)
Cardiac	2.8 (1∙.–6.7)	1.9 (0.8–4.7)	3.3 (2.3–4.8)	2.3 (1.6–3.5)	0.8 (0.1–5.9)	-	4.3 (1.1–17.5)	2.3 (0.5–9.6)
Renal	8.0 (2.9–21.7)	3.7 (1.3–10.7)	2.6 (1.2–5.6)	1.8 (0.8–3.8)	-	-	23.6 (7.4–74.4)	8.7 (2.5–30.2)
Limb	4.5 (2.0–10.1)	3.9 (1.7–9.0)	2.0 (1.1–3.4)	1.7 (0.9–3.1)	-	-	8.8 (2.8–27.6)	7.1 (2.1–23.5)
Lip/Cleft/Palate	5.1 (1.9–13.6)	4.2 (1.5–11.6)	1.4 (0.6–3.2)	1.3 (0.6–3.0)	1.9 (0.3–13.4)	2.8 (0.4–20.3)	9.9 (2.4–39.9)	8.2 (2.0–34.3)
Chromosomal	3.1 (0.7–12.3)	0.9 (0.1–6.6)	1.7 (0.8–3.9)	0.8 (0.3–2.2)	-	-	6.0 (0.8–42.9)	0.8 (0.2–4.1)

^a^OR Adjusted for Age, Education, Previous births and Marital Status with country and facility as random mixed effect variable

When adjusted for covariates and for mixed effects at country and hospital levels, we found that newborns of women with chronic maternal hypertension are at higher risk of renal (aOR 3.7, 95 % CI 1.3–10.7), limb (aOR 3.9, 95 % CI 1.7–9.0) and lip/cleft/palate malformations (aOR 4.2, 95 % CI 1.5–11.6) while preeclampsia held significant associations with “Cardiac” (aOR 2.3, 95 % CI 1.6–3.5). Eclampsia was not associated to the malformation for which data were available (Lip/Cleft/Palate).

The multivariable analysis strengthened the association between the chronic hypertension with superimposed preeclampsia and “neural tube/central nervous system” (aOR 4.3, 95 % CI 1.3–14.4), “Renal” (aOR 8.7, 95 % CI 2.5–30.2), “Limb” (aOR 7.1, 95 % CI 2.1–23.5) and “Lip/Cleft/Palate” (aOR 8.2, 95 % CI 2.0–34.3) malformations (Table 4).

The sensitivity analysis carried without the African countries showed no substantial differences on finding with the original pooled analysis (Table 5).

**Table 5:** Sensitivity Analysis: Crude and adjusted Odds Ratio of pregnancy hypertensive disorders and congenital malformations excluding AFR countries

	Chronic Hypertension		Pre-eclampsia		Eclampsia		Chronic HT/preeclampsia	
	Crude OR (CI 95 %)	Adj OR^a^ (CI 95 %)	Crude OR (CI 95 %)	Adj OR^a^ (CI 95 %)	Crude OR (CI 95 %)	Adj OR^a^ (CI 95 %)	Crude OR (CI 95 %)	Adj OR^a^ (CI 95 %)
Congenital malformation								
Neural tube/Central Nervous System	4.4 (1.8–10.7)	2.4 (0.9–6.1)	1.3 (0.6–2.7)	1.0 (0.5–2.1)	-	-	9.7 (3.1–30.5)	4.2 (1.3–14.1)
Cardiac	2.6 (1.1–6.2)	1.9 (0.8–4.8)	3.1 (2.2–4.5)	2.4 (1.6–3.6)	1.2 (0.2–8.5)	-	3.7 (0.9–15.1)	2.3 (0.5–9.6)
Renal	7.4 (2.7–20.2)	3.7 (1.3–10.7)	2.5 (1.1–5.3)	1.7 (0.8–3.8)	-	-	20.5 (6.5–64.9)	8.4 (2.4–29.5)
Limb	5.1 (2.3–11.6)	4.0 (1.7–9.5)	1.6 (0.8–3.0)	1.3 (0.7–2.6)	-	-	9.4 (3.0–29.4)	7.1 (2.1–23.5)
Lip/Cleft/Palate	5.3 (1.9–14.2)	4.4 (1.6–12.0)	1.5 (0.6–3.3)	1.4 (0.6–3.3)	3.0 (0.4–21.7)	2.9 (0.4–20.4)	9.6 (2.4–38.8)	7.7 (1.8–31.9)
Chromosomal	3.0 (0.7–12.1)	0.9 (0.1–6.7)	1.4 (0.6–3.4)	0.8 (0.3–2.3)	-	-	5.5 (0.8–39.2)	0.7 (0.3–4.3)

^a^OR Adjusted for Age, Education, Previous births and Marital Status with country and facility as random mixed effect variable

## Discussion

This multicountry, facility-based survey showed wide differences in the prevalence and ascertainment of congenital abnormalities in facility deliveries across the 29 countries with occupied Palestinian territory the leading country for almost all defects such as “Cardiac” malformations with 24.6 cases per 1000 live births. Conversely some African countries showed the lowest incidence rates like Niger reporting overall only two “Limb” malformations with an overall rate of 0.2 cases per 1000 live births (Table 1). At aggregated level, the highest rates were observed in the American Region and the lowest in Africa Region. On the other hand, higher rates of birth defects were seen in higher HDI countries showing a gradual decrease with lowering HDI. Interpretation of these patterns needs caution as better screening for congenital malformations in countries with higher HDI is likely to be directly related to higher resourced facilities which in turn would explain higher identification and reporting rates in these countries. We cannot rule out that this as a reason for the lower rates in the African Region (0.25 %).

These figures deserve a number of considerations involving accuracy and can very likely be considered underestimates. We excluded stillbirths and multiple pregnancies from the analysis despite the fact that congenital abnormalities generally accounts for a considerable proportion of all stillbirths.

In addition, although the WHOMCS was conducted mainly in secondary and tertiary facilities, and these data might not be representative of newborn outcomes and maternal conditions in smaller facilities or in the community; it is also likely that some selective ascertainment occurred in relation to maternal pathologic conditions. We also compared our results for specific malformation categories with other national sources [20] for countries such as Japan where we found no substantive differences. The 2012 Annual Report of the International Clearinghouse for Birth Defects Surveillance and Research reported between 2 and 3 lip/cleft/palate malformations per 1000 total births (livebirths and stillbirths) for Japan compared to 2.2 per 1000 livebirths of the WHOMCS.

On the other hand the true rate of cardiovascular defects is around 1 %, known from numerous studies [25, 26], whilst in this study the overall rate is only 0.16 %, again indicating a certain level of under ascertainment.

Even though our dataset does not provide specific information on consanguinity, high frequency of congenital disorders in countries of the Eastern Mediterranean Region is largely regarded as due to consanguineous marriage, 25–70 % of unions involve related family members: parental consanguinity increases the birth prevalence of autosomal recessive birth defects and the risk of neonatal and childhood death, intellectual disability and serious birth defects is almost doubled for first cousin unions [27–30].

Our analysis revealed around four times increased odds of “renal”, “limb” and “lip/cleft/palate” malformations associated with chronic maternal hypertension (Table 4); these associations were further increased in the condition of chronic hypertension with superimposed preeclampsia where adjusted odds ratio ranged from 7.1 for “limb” to 8.7 for “renal” malformations. The analysis also showed 2.3 and 1.6 times increased odd of respectively “cardiac” and “other” malformations due to preeclampsia as well as 4.3 times increased odd of “neural tube/central nervous system” malformations due to chronic hypertension with superimposed preeclampsia.

Since no correlation was detected between WHO regions with respect to registered malformations risk and registered hypertension risk the association between the two variables is likely to be true.

Pregnant women with chronic hypertension presumably had this condition prior to conception and throughout pregnancy; therefore, during the critical period for organogenesis where major congenital abnormalities can develop, it is important to remark how a “dose/effect relationship” manifests when considering the increased risk of renal, limb and lip/cleft/palate malformations due to the chronic hypertension with superimposed preeclampsia when compared with the significant associations of the same defects with only chronic hypertension. This effect has also been shown in a recent large case–control study in US although this study did not find statistically significant association between hypertensive disorders and these specific birth defects [31].

The association between chronic hypertension with superimposed preeclampsia with renal dysgenesis has been reported by a previous case–control study [12]; however we should address the lack of information in our datasets concerning use of angiotensin-converting enzyme inhibitors and angiotensin II receptor inhibitors/antagonists which are contraindicated in pregnant women due to their teratogenicity [32].

Other studies found similar effects of beta-blockers [33] and suggested that the general effect of anti-hypertensives could be an effect of drug-induced fetal hypotension or an effect of the underlying disease [34].

It is difficult to determine what possible mechanisms may have led to the association of chronic hypertension with higher rates of limb and lip/cleft/palate defects.

The lack of association between preeclampsia and most of congenital malformations can be explained by the onset of this hypertensive disorder after the first trimester; nevertheless the association of preeclampsia with cardiac defects might be explained in line with a common genetic predisposition for angiogenic/anti-angiogenic imbalance in maternal and fetal blood [35].

It also not obvious to argue a causal effect of chronic hypertension with superimposed preeclampsia on the insurgence of neural tube defects; however a recent article put the evidence on the effect of the folic acid supplementation, well known for protecting from the risk of neural tube/spina bifida [36, 37], in reducing the risk of gestational hypertension and preeclampsia [38] suggesting again a common pathway for the two conditions.

Strengths of this analysis include the large and multi-country dataset for the purpose of exploring the incidence of congenital malformation and its association with hypertensive disorders of pregnancy. Consistency in study methodology and definitions across facilities and countries enhance comparability. In addition, the large sample size allows for stratification by type of hypertensive disorder providing more clinical relevance.

This analysis presents several limitations. In the WHOMCS, a specific ascertainment of congenital malformations was not carried out and only broader groups of congenital malformations were included. Hence we were not able to consider the specific birth defect categories that may be associated with hypertensive disorders. In this regard, we were unable to assess associations between specific congenital malformations (such as esophageal atresia and hypospadias) and hypertensive disorders that have been identified in other studies [11–14]. Another limitation concerns the outcome variable, congenital malformations, being ascertained at birth or immediately after birth. Some birth defects like several cardiac lesions appear late (there may be delayed appearance/capture even up to 5 years) [17].

For the exposure variables (“Eclampsia”, “Pre-eclampsia” and “Chronic Hypertension”) time of onset or duration of exposure is not specified in WHOMCS database. This is important since many congenital malformations occur before 8 weeks of gestation and risk lies in pre-conception period. In addition, the WHOMCS did not collect data on antihypertensive medication exposure and other risk factors associated with a number of specific birth defects. Many other major risk factors (eg. diabetes, smoking, obesity, alcohol use) were not available to give adjusted OR. ORs were calculated only for age, education, previous births and marital Status.

Disparities in the ascertainment of congenital malformations in high and low HDI countries is clearly apparent with very high and high HDI countries reporting a majority of congenital malformations even with lesser number of live births.

## Conclusions

To our knowledge this is one of the few analyses reporting estimate the prevalence of congenital malformation in facilities in middle and low income countries where more than 90 % of all infants with a serious birth defect are born and where many of them will die young because of lack of adequate health care services [39]. A concerted effort to start a systematic collection, analysis and dissemination of data on congenital malformation is crucial for the years to come when the burden of disease will increase with the steady proportional decrease of post neonatal mortality. As a step towards this objective, in 2014 WHO published a manual intended to serve as a tool for the development, implementation and ongoing improvement of congenital anomalies surveillance programmes, particularly for countries with limited resources [40].

In view of findings and characteristics of our study we also suggest additional research on associations and possible common pathogenesis pathways in maternal hypertensive disorders and congenital defects, given their roles in maternal and child morbidity and mortality.

## Abbreviations

AFR, African Region; AMR, American Region; EMR, Eastern Mediterranean Region; HDI, human development index; MCS, multi-country survey; SEAR, South-East Asian Region; WHO, World Health Organization; WPR, Western Pacific Region
